# Molecular analysis and distribution of multidrug-resistant *Enterococcus faecium* isolates belonging to clonal complex 17 in a tertiary care center in Mexico City

**DOI:** 10.1186/1471-2180-13-291

**Published:** 2013-12-11

**Authors:** Sara A Ochoa, Gerardo Escalona, Ariadnna Cruz-Córdova, Leticia B Dávila, Zeus Saldaña, Vicenta Cázares-Domímguez, Carlos A Eslava, Briceida López-Martínez, Rigoberto Hernández-Castro, Guillermo Aquino-Jarquin, Juan Xicohtencatl-Cortes

**Affiliations:** 1 Departamento de Infectología, Laboratorio de Bacteriología Intestinal, Hospital Infantil de México Federico Gómez, México City, DF 06720, México; 2 Departamento de Salud Pública, Facultad de Medicina, Universidad Nacional Autónoma de México, Av. Insurgentes Sur s/n., México City, DF 04510, México; 3 Departamento Clínico, Hospital Infantil de México Federico Gómez, México City, DF 06720, México; 4 Departamento de Ecología de Agentes Patógenos, Hospital General “Dr. Manuel Gea González”, Tlalpan 14080, México; 5 Unidad de Investigación en Enfermedades Oncológicas, Hospital Infantil de México Federico Gómez, Dr. Márquez 162, Col. Doctores, Delegación Cuauhtémoc, México City, DF 06720, México

**Keywords:** *Enterococcus faecium*, Multidrug-resistant, Clonal complex, Pulsotypes, Virulence

## Abstract

**Background:**

*Enterococcus faecium* has recently emerged as a multidrug-resistant nosocomial pathogen involved in outbreaks worldwide. A high rate of resistance to different antibiotics has been associated with virulent clonal complex 17 isolates carrying the *esp* and *hyl* genes and the *purK*1 allele.

**Results:**

Twelve clinical vancomycin-resistant *Enterococcus faecium* (VREF) isolates were obtained from pediatric patients at the Hospital Infantil de México Federico Gómez (HIMFG). Among these VREF isolates, 58.3% (7/12) were recovered from urine, while 41.7% (5/12) were recovered from the bloodstream. The VREF isolates showed a 100% rate of resistance to ampicillin, amoxicillin-clavulanate, ciprofloxacin, clindamycin, chloramphenicol, streptomycin, gentamicin, rifampicin, erythromycin and teicoplanin. In addition, 16.7% (2/12) of the isolates were resistant to linezolid, and 66.7% (8/12) were resistant to tetracycline and doxycycline. PCR analysis revealed the presence of the *vanA* gene in all 12 VREF isolates, *esp* in 83.3% (10/12) of the isolates and *hyl* in 50% (6/12) of the isolates. Phylogenetic analysis via molecular typing was performed using pulsed-field gel electrophoresis (PFGE) and demonstrated 44% similarity among the VREF isolates. MLST analysis identified four different sequence types (ST412, ST757, ST203 and ST612).

**Conclusion:**

This study provides the first report of multidrug-resistant VREF isolates belonging to clonal complex 17 from a tertiary care center in Mexico City. Multidrug resistance and genetic determinants of virulence confer advantages among VREF in the colonization of their host. Therefore, the prevention and control of the spread of nosocomial infections caused by VREF is crucial for identifying new emergent subclones that could be challenging to treat in subsequent years.

## Background

Enterococci are opportunistic pathogens of the normal intestinal microbiota of humans and animals [1,2]. The most common species of *Enterococcus* involved in nosocomial infections is *Enterococcus faecium* (*E. faecium*) [1,2]. This pathogen is associated with hospital-acquired infections such as UTIs (urinary tract infections), wounds, bacteremia, endocarditis and meningitis [1,2].

In recent years, the emergence of multidrug-resistant *E. faecium* has increased [3-5]. The recommended treatment for *Enterococcus* infections has been penicillin alone or combined with aminoglycosides. However, due to increased resistance to aminoglycosides, vancomycin is currently the antibiotic employed to treat these infections. In the last several decades, the number of vancomycin-resistant enterococci (VRE) has increased. The first VRE isolates were reported in the United Kingdom in the late 1980s [6]. In the United States, more than 80% of *E. faecium* isolates from hospitals are now resistant to vancomycin, and virtually all of them (>90%) exhibit ampicillin resistance [7]. Vancomycin-resistant *Enterococcus faecium* (VREF) has been associated with outbreaks in hospitals worldwide [2]. The rates of VREF colonization and infection have risen steadily, with most cases being caused by strains displaying glycopeptide resistance to VanA and VanB [8-11].

In addition to multidrug resistance, *E. faecium* produces diverse factors that contribute to its pathogenesis, including virulence molecules such as secreted antigen SagA [12], cell wall-anchored collagen adhesin (Acm) [13], hyaluronidase (Hyl) [14] and enterococcal surface protein (Esp) [15]. However, the traits that contribute to the transition of *E. faecium* from a commensal to a nosocomial pathogen have not been identified [16].

Molecular typing methods are essential for identifying hospital-associated outbreaks of *E. faecium*. Multilocus sequence typing (MLST) has revealed the existence of host-specific genogroups, including a specific genetic lineage designated clonal complex 17, associated with hospital-related isolates [1,17]. MLST of *E. faecium* is based on identifying alleles from DNA sequences in internal fragments of housekeeping genes (*atpA, ddl*, *gdh*, *purK, gyd, pstS* and *adk*), resulting in a numeric allelic profile, with each profile then being assigned a sequence type (ST) [17].

Complex 17 most likely evolved from the primary *E. faecium* ancestor ST-22, while ST-17 represents an important secondary founder with additional linages designated to complex 17 [18]. Clonal complex 17 is characterized by ampicillin and quinolone resistance and the presence of a putative pathogenicity island that includes the *esp* and/or *hyl* genes in the majority of isolates [1,18-20]. Various STs belonging to clonal complex 17, such as ST16, ST17, ST18, ST203 and ST412, are currently being disseminated worldwide [21,22]. Interestingly, half of the STs within the clonal complex 17 polyclonal subpopulation have also been identified in samples obtained from healthy humans, swine, poultry and pets [16].

In Mexico, there is little available information about the prevalence of VREF isolates, and no study related to clonal complex 17 has been performed in pediatric patients. The aim of this study was to genotypically and phenotypically characterize VREF clinical isolates from 12 immunocompromised pediatric patients at the Hospital Infantil de México Federico Gómez (HIMFG). This study involved amplification of the resistance genes *vanA* and *vanB* and two virulence genes (*esp* and *hyl*) and molecular typing via pulsed-field gel electrophoresis (PFGE) and MLST.

## Methods

### Bacterial isolates

Twelve *E. faecium* isolates of clinical importance were obtained from 12 patients with nosocomial infections in the PICU (Pediatric Intensive Care Unit), oncology, gastroenterology and transplant wards of HIMFG during the period from July 2009 to April 2011. The isolates were maintained at −70°C in skim milk (Becton Dickinson, New Jersey, USA) and cultured on 5% sheep blood agar plates (Becton Dickinson, New Jersey, USA) at 37°C under 5% CO_2_ for 24 h. The *E. faecalis* ATCC® 29212, *E. faecalis* ATCC® 51299 and *E. faecium* ATCC® 51559 strains (American Type Culture Collection Manassas, VA, USA) were used as controls.

### Biochemical tests

Bacteria were grown on blood agar, and identification was performed using manual methods. All colonies were grown in brain heart infusion broth (BHI) (Becton Dickinson, New Jersey, USA) with 6.5% NaCl and on bile esculin agar (Oxoid Sunnyvale, California, USA) to determine their hydrolysis grade. Disks impregnated with the substrate L-pyrrolidonyl-beta-naphthylamide were used to perform pyrrolidonase tests (Oxoid Biochemical Identification System, Oxoid LTD., Basingstoke, Hampshire, England). Reduction of tellurite (Merck, Darmstadt, Germany) was evaluated via growing the bacteria on 0.04% potassium tellurite.

### Antibiotic susceptibility

The antibiotic susceptibility profiles of the 12 VREF isolates were determined via the minimum inhibitory concentration (MIC) technique by means of the microdilution method using Mueller-Hinton broth (MHB), as recommended by the Clinical and Laboratory Standards Institute. MIC tests were performed for vancomycin (MP Biomedicals, Solon, Ohio, USA), teicoplanin (Sigma-Aldrich, St. oLouis, Missouri, USA), chloramphenicol (MP Biomedicals, Solon, Ohio, USA), ciprofloxacin (MP Biomedicals, Solon, Ohio, USA), streptomycin (Alexis Biochemical, San Diego California, USA), linezolid (Sigma-Aldrich, St. Louis, Missouri, USA), rifampicin (MP, Biomedicals, Ohio, USA), nitrofurantoin (MP Biomedicals, Solon, Ohio, USA), tetracycline (MP Biomedicals, Solon, Ohio, USA), doxycycline (Sigma-Aldrich, St. Louis, Missouri, USA), erythromycin (MP Biomedicals, Solon, Ohio, USA), tigecycline (Sigma-Aldrich, St. Louis, Missouri, USA), gentamicin (MP Biomedicals, Solon, Ohio, USA) and amoxicillin-clavulanate (Glaxo-Smith-Kline, Philadelphia, Pennsylvania, USA). Several concentrations (256–0.625 μg/ml) of the antibiotics were tested in Mueller Hinton broth, with 100 μl of those dilutions being loaded into each well of a microplate. For each dilution, 100 μl of a bacterial suspension (1.5x10^8^ CFU/ml) was inoculated and grown overnight at 37°C under a CO_2_ atmosphere. After bacterial growth was detected, the MIC for each isolate of *E. faecium* was reported as the highest concentration (μg/ml) of antibiotics in which no growth was observed. The *E. faecalis* ATCC® 29212 strain (American Type Culture Collection Manassas, VA, USA) was used as a control. These isolates were also evaluated for high-level aminoglycoside resistance (HLAR) to streptomycin (1,000 μg/ml) and gentamicin (500 μg/ml).

### Detection of the glycopeptide resistance genes *vanA* and *vanB*

PCR was performed to detect the glycopeptide resistance genes *vanA* and *vanB* in the 12 *E. faecium* clinical isolates using specific primers (Table 1) [23]. Briefly, genomic DNA was purified using the Wizard Genomic DNA Purification Kit (Promega Madison, Wisconsin, USA) from a bacterial culture grown in BHI broth incubated at 37°C for 24 h. The amplification reactions were prepared in a final volume of 50 μl, as follows: 25 μl of amplification mix (22 mM Tris/HCl, pH 8.4; 55 mM KCl; 1.65 mM MgCl_2_; 25 μM each dNTP; 0.6 U recombinant Taq DNA polymerase/ml), 100 ng/μl of bacterial DNA, 10 μl of H_2_O and 5 μl of primer solution (10 pg/μl). A Perkin Elmer 9600 thermocycler was programmed to run for 30 cycles with the following parameters: denaturing at 94°C for 3 m, annealing at 55°C for 45 s and extension at 72°C for 1 m, with a final extension at 72°C for 2 m. The samples were analyzed via electrophoresis in 1% agarose gels (Agarose LE, Promega) using a 100 bp DNA ladder (Gibco/BRL Life Technologies, Breda, The Netherlands). *E. faecium* strain ATCC 51559 (*vanA*^*+*^) and *E. faecalis* strain ATCC® 51299 (*vanB*^*+*^) were used as controls in the PCR experiments [24].

**Table 1:** Primers sequences used in this study

**Gene**	**Primer**	**Sequence (5′ to 3′)**	**Size (bp)**	**Reference**
*vanA*	vanA-F	CATGAATAGAATAAAAGTTGCAATA	1,030	(Clark et al., 1993) [23]
	vanA-R	CCCCTTTAACGCTAATACGATCAA		
*vanB*	vanB-F	GTCACAAACCGGAGGCGAGGA	433	(Clark et al., 1993) [23]
	vanB-R	CCGCCATCCTCCTGCAAAAAA		
*esp*_*Efm*_	esp-F	TTGCTAATGCTAGTCCACGACC	945	(Shankar et al., 1999) [25]
	esp-R	GCGTCAACACTTGCATTGCCGA		
*hyl*_*Efm*_	hyl-F	GAGTAGAGGAATATCTTAGC	661	(Rice et al., 2003) [14]
	hyl-R	AGGCTCCAATTCTGT		

### PCR screening for the *esp* and *hyl* genes

DNA from bacterial cultures was extracted and amplified via PCR using primers for the *esp*_*Efm*_ and *hyl*_*Efm*_ genes (Table 1), generating bands of 954 bp and 661 bp, respectively [14,25].

### Molecular typing of VREF

PFGE of the 12 VREF clinical isolates was carried out following the protocols of Morrison et al. [26,27]. Briefly, the samples were digested with 50 U of SmaI (New England Biolab, Ipswich, MA, USA) for 4 h at 25°C. The digested plugs were separated via electrophoresis in 1% agarose gels (BioRad, Hercules, California, USA) using ultra-pure DNA agarose (BioRad, Hercules, California, USA), with 0.5X TBE as the running buffer in the CHEF MAPPER system (BioRad Laboratories, Hercules, California, USA), run at 6 V/cm at 14°C under two different linear ramped pulse times: 1 to 10 s for 16 h and 10 to 40 s for 22 h. A PFGE lambda ladder (New England Biolabs, Hertfordshire, England, UK) was used as a molecular weight marker, and the gels were stained for 40 m with 0.5 mg/ml of ethidium bromide for visualization under UV light. The obtained banding patterns were initially interpreted via visual inspection according to the criteria specified by Tenover et al. [28]. Cluster analysis was performed with BioNumerics (Applied Maths, Inc., Austin, TX, USA) using the DICE correlation coefficient and the unweighted pair group mathematical average algorithm (UPGMA) as the grouping method [29].

The PFGE pulsotypes of the 12 VREF clinical isolates were also genotyped through multilocus sequence typing (MLST) according to a standard protocol described by Homan et al. [17]. Fragments of seven housekeeping genes (*atpA*, *ddl*, *gdh*, *purK*, *gyd*, *pstS* and *adk*) were sequenced using a 3730xl DNA Analyzer (Applied Biosystems, Foster City, California, USA), thus obtaining their allelic profiles, and the STs for each unique allelic profile were designated on the basis of information from the MLST website (http://efaecium.mlst.net).

## Results

### Origin of the strains

A total of 12 VREF clinical isolates obtained during the period from July 2009 to April 2011 were included in this study. The risk factors of the 12 patients were characterized by a minimum hospital stay of 4 days, assistance in the PICU and treatment with vancomycin. During their stay, the 12 patients were subjected to surgical procedures and received a central venous catheter, steroids and immunosuppressive treatment. Among the VREF isolates, 58.3% (7/12) were obtained from urine, while 41.6% (5/12) were obtained from the bloodstream. The VREF isolates were obtained from patients with different pathologies (Table 2).

**Table 2:** Characteristics of the 12 VREF isolates related to the patients’ clinical diagnosis, source of clinical samples, ward, PFGE, sequence type and clonal complex

**Clinical isolate**	**Clinical diagnosis**	**Sources of clinical samples**	**Wards**	**PFGE**	**MLST/STs**	**CC**
133H	Acute lymphocytic leukemia L1, fever, and neutropenia	Bloodstream	ONC	A	757	
926U	Aplastic anemia, neutropenic colitis, septic shock	Urine	ONC	A	203	17
821U	Lupus erythematosus, septic Shock	Urine	TRPU	A	412	17
851H	Anaplastic lymphoma, tumor lysis syndrome, sepsis	Bloodstream	PICU	B	757	
215H	Venous catheter infection, Down syndrome	Bloodstream	PICU	B	612	17
222U	Acute myeloid leukemia M2, tumor lysis syndrome, Septic shock	Urine	ONC	B	412	17
127U	Acute lymphocytic leukemia L1, fever, and neutropenia.	Urine	PICU	B1	412	17
30H	Wilms tumor	Bloodstream	PICU	B1	412	17
634U	Septic shock, hemophagocytic lymphohistiocytosis	Urine	ONC	C	757	
459U	Lupus erythematosus, sacroiliac ulcers	Urine	PICU	C	412	17
422H	Acute myeloid leukemia M4, fever, and neutropenia	Bloodstream	SS	D	412	17
155U	cholestatic syndrome, choledochal cyst.	Urine	GST	D	203	17

Multilocus sequence typing (MLST), sequence types (STs), clonal complex (CC). ONC (Oncology Ward), TRPU (Transplant Unit), PICU (Pediatric Intensive Care Unit), SS (Short Stay Ward) and GST (Gastroenterology Ward).

### Detection of susceptibility patterns and glycopeptide resistance in the VREF isolates

The results obtained for the 12 VREF clinical isolates showed a 100% rate of resistance to ampicillin, amoxicillin-clavulanate, ciprofloxacin, clindamycin, chloramphenicol, streptomycin, gentamicin, rifampicin, erythromycin and teicoplanin. The MIC values for each VREF isolate are presented in Table 3. In addition, 16.7% (2/12) of the VREF clinical isolates were resistant to linezolid, and 67% (8/12) were resistant to tetracycline and doxycycline (Table 3). However, all of the VREF isolates were susceptible to nitrofurantoin and tigecycline (Table 3). The HLAR values for gentamicin (500 μg/ml), streptomycin (1,000 μg/ml) and gentamicin/streptomycin (500/1,000 μg/ml) were determined with to 50% (6/12), 25% (3/12) and 25% (3/12), respectively.

**Table 3:** **Minimum Inhibitory Concentration (MIC) to 12 clinical isolates of multidrug-resistant *Enterococcus faecium***

**Clinical isolate**	**Antibiotics (MIC μg/ml)**															
	**Am**	**Amc**	**CIP**	**CC**	**C**	**GM**	**S**	**RA**	**E**	**Va**	**TEI**	**Te**	**D**	**LZN**	**F/M**	**TGC**
133H	128	128	512	128	64	32	512	4	32	512	16	1	1	1	8	2
926U	256	256	4	128	32	32	64	4	32	512	32	64	8	2	2	2
821U	128	128	256	256	32	32	32	32	32	256	128	64	64	2	16	1
851H	128	128	512	256	32	32	≥512	4	32	512	16	2	1	4	16	2
215H	128	128	512	256	32	32	≥512	4	32	512	16	2	0.25	1	4	1
222U	64	128	256	256	32	32	32	16	32	256	32	64	16	2	32	1
127U	128	128	256	256	32	32	32	32	32	256	64	64	16	8	32	2
30H	128	128	256	256	32	32	32	16	32	256	256	64	16	1	16	2
634U	64	64	256	256	32	32	≥512	4	32	256	16	4	0.5	2	8	2
459U	256	256	256	256	64	32	32	16	32	256	32	64	16	2	16	1
422H	128	128	256	256	32	32	32	8	32	256	64	64	8	2	16	1
155U	128	128	256	256	32	32	128	8	32	256	64	64	16	2	16	2
CVR^*^	**≥16**	**≥8**	**≥4**	**≥4**	**≥32**	**≥16**	**≥16**	**≥4**	**≥8**	**≥32**	**≥16**	**≥16**	**≥8**	**≥4**	**≥128**	**≥16**
**% R**	**100**	**100**	**100**	**100**	**100**	**100**	**100**	**100**	**100**	**100**	**100**	**67**	**67**	**16.7**	**0**	**0**

Ampicillin (Am), amoxacillin/clavulanate (Amc), ciprofloxacin (CIP), clindamycin (CC), chloramphenicol (C), gentamicin (GM), streptomycin (S), rifampin (RA), erythromycin (E), vancomycin (Va), teicoplanin (TEI), tetracycline (Te), doxycycline (D), linezolid (LZN), nitrofurantoin (F/M), and tigecycline (TGC), *Cut-off values for resistance to MIC(μg/ml), Percentage of resistant (%R).

The *vanA* and *vanB* genes of the 12 VREF clinical isolates were amplified via PCR. Interestingly, only the *vanA* gene was detected in all the VREF clinical isolates, as a 1,030 bp amplicon (data not shown), whereas the *vanB* gene, with a length of 433 bp, was not identified in the isolates (data not shown). The *E. faecium* ATCC® 51559 (*vanA*^*+*^) and *E. faecalis* ATCC® 51299 (*vanB*^*+*^) strains were used as positive controls in the PCR assays [24].

### Prevalence of the *esp* and *hyl* virulence genes in the VREF isolates

The *esp* and *hyl* virulence genes, which are associated with a clonal subcluster known as clonal complex 17 in VREF clinical isolates, were detected via PCR. The *esp* and *hyl* genes were highly prevalent in the isolates. The *esp* virulence gene was detected in 83.3% (10/12) of the isolates, and the *hyl* virulence gene was present in 50% (6/12) of them. Therefore, three genotypes were determined for the VREF clinical isolates: *esp*^*+*^*/hyl*^*-*^, *esp*^*+*^/*hyl*^*+*^ and *esp*^*-*^/*hyl*^*+*^, at prevalence rates of 50% (6/12), 33.3% (4/12) and 16.7% (2/12), respectively.

### Molecular typing analysis of the *E. faecium* isolates via PFGE and MLST

**Figure 1:**
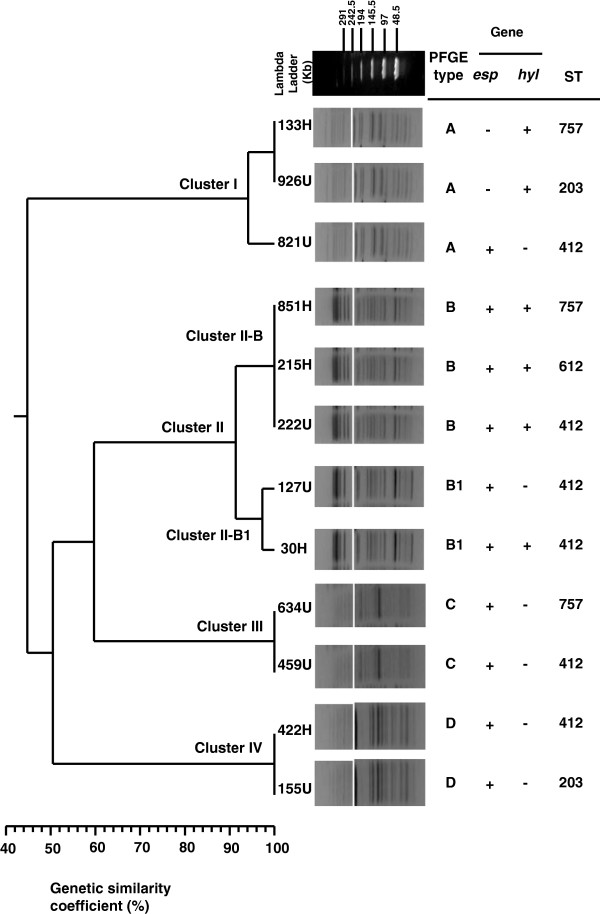
**PFGE analysis of 12 VREF isolates recovered at HIMFG and detection of the virulence factors *esp *and *hyl*, sequence type, isolation ward and type of sample.** Phylogenetic analysis was performed using the DICE coefficient in association with the UPGMA algorithm as the grouping method. The dendrogram was evaluated by obtaining the cophenetic correlation coefficient using the Mantel test, which yielded an r value of 0.97769.

The VREF isolates were analyzed via PFGE following SmaI digestion of genomic DNA. Data obtained through PFGE were analyzed using a dendrogram profile, which included the PFGE pulsotypes obtained from VREF (Figure 1). A total of four clusters (I-IV) with five DNA pulsotypes were identified, showing patterns consisting of 12 to 20 DNA fragments ranging in size from 48.5 to 339.5 Kb (Figure 1). Interestingly, 25% (3/12) of the VREF clinical isolates observed via PFGE were categorized as pulsotype B and 16.7% (2/12) as pulsotype B1, with 92% genetic similarity being observed among these isolates (Figure 1). Meanwhile, 25% (3/12) of the VREF isolates were classified as pulsotype A, showing a different pattern from pulsotypes B, C and D (Figure 1). However, 16.7% (2/12) of the VREF isolates were classified as pulsotypes C and D, which displayed 50% genetic similarity. In addition, a maximum of 44% similarity was observed among all clusters of VREF isolates.

**Figure 2:**
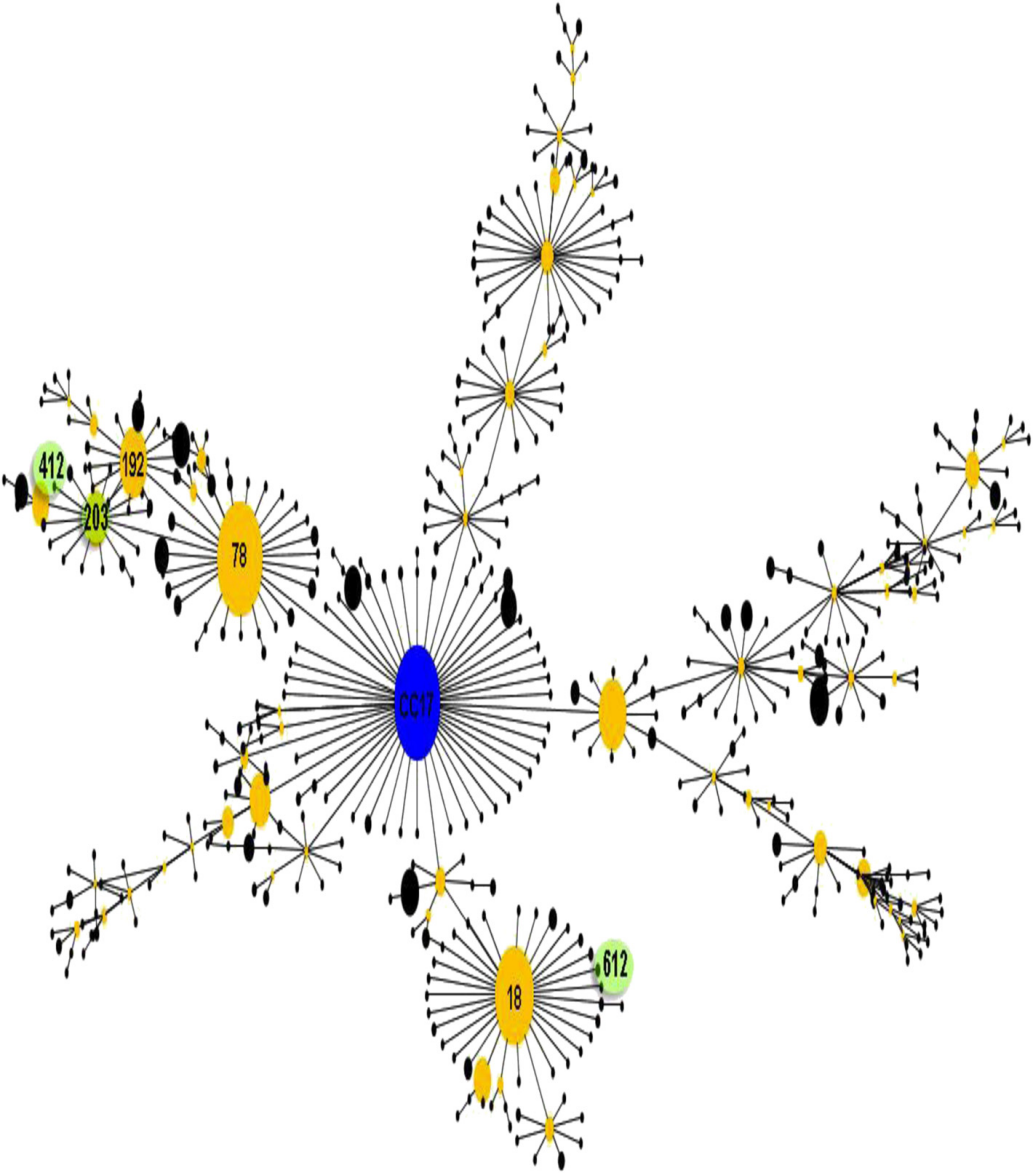
**Clustering of MLST profiles using the eBURST database algorithm.** Our profiles showed that ST412, ST612 and ST203, but not ST757, belong to clonal complex 17.

In this study, 12 VREF clinical isolates were subjected to MLST genotyping. Six of the 12 VREF isolates (50%) belonged to ST412, three to ST757, two to ST203 and one to ST612 (Table 2). eBURST analysis of the VREF isolates revealed four different STs (ST412, ST612, ST757 and ST203), three of which belonged to clonal complex 17; ST757 was not related to this clonal complex (Figure 2).

## Discussion

*E. faecium* is a highly resistant nosocomial pathogen and has recently emerged as an important threat in hospitals worldwide [2]. In this study, the 12 examined VREF isolates exhibited multidrug resistance to ampicillin, amoxicillin-clavulanate, ciprofloxacin, clindamycin, chloramphenicol, streptomycin, gentamicin, rifampicin, erythromycin and teicoplanin. At HIMFG, several types of enterococcal infections in pediatric patients are commonly treated with a combination of drugs (aminoglycoside-β-lactams, such as gentamicin/ampicillin) as the first choice, while vancomycin is the second choice; vancomycin-aminoglycoside or linezolid is the third choice; and tigecycline is the fourth choice. Interestingly, 16.7% (2/12) of the VREF clinical isolates were also resistant to linezolid, and 67% (8/12) were resistant to both tetracycline and doxycycline. The emergence of high levels of resistance to the most common anti-enterococcal antibiotics (vancomycin) might constitute a real challenge in the treatment of these infections. In the present study, 100% (12/12) of the examined VREF isolates were susceptible to tigecycline and nitrofurantoin. The VREF resistance patterns observed in this study are in agreement with the findings of other authors [30,31]. However, these authors observed VREF isolates that were susceptible to linezolid and nitrofurantoin, in contrast to our data, which showed that two of the VREF isolates were resistant to linezolid. Nevertheless, the low resistance to linezolid observed in the VREF clinical isolates is in accord with data reported in other countries [11,32].

Few instances of the isolation of HLAR *E. faecium* have been documented worldwide [22,33,34]. In this study, the examined VREF clinical isolates showed HLAR to gentamicin (500 μg/ml), streptomycin (1,000 μg/ml) and gentamicin/streptomycin (500/1,000 μg/ml), displaying resistance values of 50%, 25% and 25%, respectively. Treatment of severe enterococcal infection requires combined therapy to achieve a synergistic bactericidal effect [35]. However, the results obtained in cases of severe infections associated with enterococci have shown that HLAR should not be treated with combined therapy (gentamicin/ampicillin) [35]. Therefore, the treatment of HLAR *E. faecium* is restricted [36].

The enterococcal surface protein Esp, which is encoded by genes that appear to have been acquired and localized within a pathogenicity island, is commonly found in clinical isolates and anchors to the cell wall. This protein also affects biofilm formation and plays a role in experimental UTI and/or endocarditis models [2]. The presence of the *esp* gene has been associated with hospital outbreaks, although this gene is not exclusively found in epidemic strains [19,30,37,38]. The *esp* gene was detected in 83.3% of our VREF clinical isolates. In addition, the majority of *esp*^*+*^ strains of *E. faecium* isolates were multidrug-resistant to more than three antibiotics, in accord with data reported by other researchers [39-41].

On the other hand, the *hyl* gene was found in 50% of the VREF clinical isolates and displayed a higher prevalence compared to the prevalences of 29.8% (29/131) reported in isolates of *E. faecium* in the Picardy Region of France, 38% (83/220) in isolates from the US and 3% in European clinical isolates. However, in the United Kingdom, a *hyl* gene prevalence of 71% (20/28) was observed in *E. faecium* isolates [14,42,43]. We believe that the differences observed in the detection rates of the *hyl* gene are due to the region in which the samples were isolated. The rates of the occurrence of *esp*^+^/*hyl*^-^, *esp*^+^/*hyl*^+^ and *esp*^-^/*hyl*^+^ isolates were found to be 50% (6/12), 33.3% (4/12) and 16.7% (2/12), respectively, which is in accord with the findings of Vankerckhoven et al. and Rice et al. [14,42,44]. The VREF clinical isolates of Mexican origin in which the *esp* and/or *hyl* gene was amplified (alone or together), were resistant to more than three antibiotics; in contrast, other studies have shown a significant correlation between the presence of the *esp* gene and resistance to ampicillin, imipenem and ciprofloxacin [40,41].

PFGE and MLST analyses have been proposed as alternative methods for the molecular characterization of clinical isolates of *E. faecium*[45]. According to our PFGE analysis, the 12 VREF isolates showed a heterogeneous pattern associated with a profile of multidrug resistance to different antibiotics and the presence of the *vanA* gene. The data obtained through PFGE revealed four clusters (I-IV), with a low similarity of 44% being detected among the VREF isolates and therefore high diversity. Furthermore, the VREF isolates within clusters I, II-B and III showed an identical banding profile in the PFGE analysis. However, the MLST data indicated different STs due to changes in the nucleotide sequences of the analyzed housekeeping genes; these data are consistent with the findings of Poh et al. [46]. In addition, the VREF isolates within clusters II-B1 and IV displayed identical PFGE and MLST profiles, in agreement with other authors [22,33]. Nevertheless, pulsotypes from different wards showed similar multidrug resistance profiles, possibly due to horizontal genetic transference between these isolates.

MLST is an important tool for studying the molecular epidemiology of outbreaks of *E. faecium* and microbial population biology [44]. MLST analysis of VREF clinical isolates revealed four STs: ST203, ST412, ST612 and ST757. As previously reported, clonal complex 17 harbors various STs that have been involved in hospital outbreaks. Our results revealed two allelic profiles, ST203 and ST412, belonging to clonal complex 17 STs involved in hospital outbreaks. However, clonal complex 17 has been resolved into two different subgroups, one of which harbors ST17 and ST18, while the second harbors ST78 [47]. ST17, ST18 and ST203 are the major groups in the genetic lineage of *E. faecium*; they are distributed worldwide and have been associated with outbreaks [18,48]. ST412 was the most frequent sequence type found in the VREF isolates from HIMFG and was genetically linked to the ST78 lineage. Interestingly, ST412 has been identified worldwide and associated with outbreaks [49]. According to the eBURST analysis, ST612 showed characteristics of the STs belonging to the 18 lineage. ST757 has not been characterized within clonal complex 17. In addition, ST757 displayed resistance markers (ampicillin and quinolones), virulence genes (*esp*^+^ and/or *hyl*^+^) and the *purK1* allele; however, it has not been associated with outbreaks. Nevertheless, this community of multidrug-resistant strains is able to infect humans and might contribute to the spreading of these bacteria in the hospital, highlighting the importance of molecular typing via MLST to identify STs involved in nosocomial outbreaks.

Recently, it was shown that MLST analysis of typified *E. faecium* based on selected alleles may generate misleading results due to the recombination of five alleles (*atpA, ddl*, *gdh*, *gyd* and *pstS*). As only the *purk* and *adk* alleles are located in regions where there is no predicted recombination, the results must be interpreted with care [50]. The genome of *E. faecium* is highly plastic due to the few existing barriers to the acquisition of foreign genetic elements [51,52]. Recent studies have provided evidence of high levels of recombination through comparative genomics analyses [51-54]. Whole-genome sequencing platforms are superior to conventional typing methods, providing an excellent tool for determining phylogenies and regions of recombination and for accurately discriminating between outbreak- and non-outbreak-causing VREF isolates [50,55]. Thus, whole-genome sequence information, rather than data on just one or a few genes, could be used to distinguish between closely related strains.

In this study, MLST and PFGE analysis were applied for the molecular characterization of clinical VREF isolates to identify different clonal complexes with different pulsotypes that were not related to outbreaks. However, according to the results obtained through PFGE, four multidrug-resistant clones of VREF were identified at HIMFG; in addition, these VREF isolates were identified at different periods. Therefore, these data suggest that these clones have circulated endemically at HIMFG.

In the case of cluster II, the clones have evolved from cluster II-B to cluster II-B1 due to the high similarity (> 90%) observed via PFGE analysis and based on the acquisition of three bands for B1, suggesting a mechanism of horizontal gene transfer. The results obtained in this study highlight the importance of monitoring circulating VREF isolates in different wards of this institution to efficiently control multidrug resistance and prevent outbreaks of these clones.

## Conclusion

Little is known about VREF isolates in Mexican hospitals. In this study, the detected virulence genes (*esp* and *hyl*), multidrug profiles and allelic patterns were associated with clonal complex 17 VREF clinical isolates obtained from pediatric patients at HIMFG. To our knowledge, this is the first report describing clonal complex 17 VREF isolates in a tertiary care center in Mexico City.

Multidrug resistance and genetic determinants of virulence confer advantages in VREF in the colonization of their hosts. The genome of *E. faecium* is highly plastic, showing an ability to readily acquire genes involved in environmental persistence, colonization and virulence, favoring the selection of specific clonal complexes in a hospital environment. Therefore, the prevention and control of the propagation of nosocomial infections caused by VREF is crucial for identifying new emergent subclones that could be challenging to treat in subsequent years.

### Ethics statement

The study was reviewed and approved by the Research (Dr. Onofre Muñoz Hernández), Ethics (Dr. Amparo Faure Fontenla) and Biosecurity (Dr. Herlinda Vera Hermosillo) Committee of HIMFG, under permit numbers HIM/2011/019. After looking at the medical history of each patient, *E. faecium* isolates were recovered from clinical samples, and the patients were asked by the physicians in the Infectology Department of HIMFG for their permission for their samples be used in this study. Analyses of *E. faecium* isolates obtained from clinical samples are not considered routine studies. Informed consent was obtained from the patient for the publication of this report and any accompanying images.

## Abbreviations

Am: Ampicillin; Amc: Amoxacillin/clavulanate; CIP: Ciprofloxacin; CC: Clindamycin; C: Chloramphenicol; GM: Gentamicin; S: Streptomycin; RA: Rifampin; E: Erythromycin; Va: Vancomycin; TEI: Teicoplanin; Te: Tetracycline; D: Doxycycline; LZN: Linezolid; F/M: Nitrofurantoin; TGC: Tigecycline; Hyl: *E. faecium* hyaluronidase; Esp: Enterococcal surface protein.

## Competing interests

The authors declare that they have no competing interests.

## Authors’ contributions

SAO, LBD and GE performed the susceptibility pattern analysis, molecular genetics experiments and PFGE and MLST assays. SAO, ZS and ACC participated in editing the manuscript and the data analysis. VCD, CAE, BLM, RHC and GAJ conducted the diagnoses of the patients, interpreted data, collaborated in the collection of samples and revised the manuscript. JXC is the principal investigator and conceived the study, designed the experiments, performed data analysis and wrote the manuscript. All authors read and approved the final version.

## References

[B1] Top J Willems R Bonten M Emergence of CC17 *Enterococcus faecium*: from commensal to hospital-adapted pathogen FEMS Immunol Med Microbiol 2008 13 3 297 308 10.1111/j.1574-695X.2008.00383.x 18279340

[B2] Arias CA Murray BE The rise of the *Enterococcus*: beyond vancomycin resistance Nat Rev Microbiol 2012 13 4 266 278 10.1038/nrmicro2761 22421879 PMC3621121

[B3] Grayson ML Eliopoulos GM Wennersten CB Ruoff KL De Girolami PC Ferraro MJ Moellering RC Jr Increasing resistance to beta-lactam antibiotics among clinical isolates of *Enterococcus faecium*: a 22-year review at one institution Antimicrob Agents Chemother 1991 13 11 2180 2184 10.1128/AAC.35.11.2180 1803989 PMC245356

[B4] Jones RN Sader HS Erwin ME Anderson SC Emerging multiply resistant enterococci among clinical isolates. I. Prevalence data from 97 medical center surveillance study in the United States. *Enterococcus* Study Group Diagn Microbiol Infect Dis 1995 13 2 85 93 10.1016/0732-8893(94)00147-O 7628198

[B5] Rice LB Emergence of vancomycin-resistant enterococci Emerg Infect Dis 2001 13 2 183 187 10.3201/eid0702.010205 11294702 PMC2631700

[B6] Uttley AH Collins CH Naidoo J George RC Vancomycin-resistant enterococci Lancet 1988 13 8575–6 57 58 2891921 10.1016/s0140-6736(88)91037-9

[B7] Hidron AI Edwards JR Patel J Horan TC Sievert DM Pollock DA Fridkin SK NHSN annual update: antimicrobial-resistant pathogens associated with healthcare-associated infections: annual summary of data reported to the national healthcare safety network at the centers for disease control and prevention, 2006–2007 Infect Control Hosp Epidemiol 2008 13 11 996 1011 10.1086/591861 18947320

[B8] Coque TM Tomayko JF Ricke SC Okhyusen PC Murray BE Vancomycin-resistant enterococci from nosocomial, community, and animal sources in the United States Antimicrob Agents Chemother 1996 13 11 2605 2609 8913473 10.1128/aac.40.11.2605 PMC163584

[B9] Rice LB Antimicrobial resistance in gram-positive bacteria Am J Med 2006 13 6 S11-19 S62-70 16735146 10.1016/j.amjmed.2006.03.012

[B10] Johnson AP Uttley AH Woodford N George RC Resistance to vancomycin and teicoplanin: an emerging clinical problem Clin Microbiol Rev 1990 13 3 280 291 2143434 10.1128/cmr.3.3.280 PMC358160

[B11] Deshpande LM Fritsche TR Moet GJ Biedenbach DJ Jones RN Antimicrobial resistance and molecular epidemiology of vancomycin-resistant enterococci from North America and Europe: a report from the SENTRY antimicrobial surveillance program Diagn Microbiol Infect Dis 2007 13 2 163 170 10.1016/j.diagmicrobio.2006.12.022 17368801

[B12] Teng F Kawalec M Weinstock GM Hryniewicz W Murray BE An *Enterococcus faecium* secreted antigen, SagA, exhibits broad-spectrum binding to extracellular matrix proteins and appears essential for *E. faecium* growth Infect Immun 2003 13 9 5033 5041 10.1128/IAI.71.9.5033-5041.2003 12933846 PMC187350

[B13] Nallapareddy SR Singh KV Murray BE Contribution of the collagen adhesin Acm to pathogenesis of *Enterococcus faecium* in experimental endocarditis Infect Immun 2008 13 9 4120 4128 10.1128/IAI.00376-08 18591236 PMC2519397

[B14] Rice LB Carias L Rudin S Vael C Goossens H Konstabel C Klare I Nallapareddy SR Huang W Murray BE A potential virulence gene, *hylEfm*, predominates in *Enterococcus faecium* of clinical origin J Infect Dis 2003 13 3 508 512 10.1086/367711 12552437

[B15] Heikens E Bonten MJ Willems RJ Enterococcal surface protein Esp is important for biofilm formation of *Enterococcus faecium* E1162 J Bacteriol 2007 13 22 8233 8240 10.1128/JB.01205-07 17827282 PMC2168697

[B16] Willems RJ Van Schaik W Transition of *Enterococcus faecium* from commensal organism to nosocomial pathogen Future Microbiol 2009 13 9 1125 1135 10.2217/fmb.09.82 19895216

[B17] Homan WL Tribe D Poznanski S Li M Hogg G Spalburg E Van Embden JD Willems RJ Multilocus sequence typing scheme for *Enterococcus faecium* J Clin Microbiol 2002 13 6 1963 1971 10.1128/JCM.40.6.1963-1971.2002 12037049 PMC130786

[B18] Willems RJ Top J Van Santen M Robinson DA Coque TM Baquero F Grundmann H Bonten MJ Global spread of vancomycin-resistant *Enterococcus faecium* from distinct nosocomial genetic complex Emerg Infect Dis 2005 13 6 821 828 10.3201/1106.041204 15963275 PMC3367597

[B19] Leavis H Top J Shankar N Borgen K Bonten M Van Embden J Willems RJ A novel putative enterococcal pathogenicity island linked to the *esp* virulence gene of *Enterococcus faecium* and associated with epidemicity J Bacteriol 2004 13 3 672 682 10.1128/JB.186.3.672-682.2004 14729692 PMC321477

[B20] Bonten MJ Willems R Weinstein RA Vancomycin-resistant enterococci: why are they here, and where do they come from? Lancet Infect Dis 2001 13 5 314 325 10.1016/S1473-3099(01)00145-1 11871804

[B21] Damani A Klapsa D Panopoulou M Spiliopoulou I Pantelidi K Malli E Kolonitsiou F Grapsa S Alepopoulou E Frantzidou F A newly described vancomycin-resistant ST412 *Enterococcus faecium* predominant in Greek hospitals Eur J Clin Microbiol Infect Dis 2010 13 3 329 331 10.1007/s10096-009-0847-9 20016994

[B22] Panesso D Reyes J Rincon S Diaz L Galloway-Pena J Zurita J Carrillo C Merentes A Guzman M Adachi JA Molecular epidemiology of vancomycin-resistant *Enterococcus faecium*: a prospective, multicenter study in South American hospitals J Clin Microbiol 2010 13 5 1562 1569 10.1128/JCM.02526-09 20220167 PMC2863891

[B23] Clark NC Cooksey RC Hill BC Swenson JM Tenover FC Characterization of glycopeptide-resistant enterococci from U.S. hospitals Antimicrob Agents Chemother 1993 13 11 2311 2317 10.1128/AAC.37.11.2311 8285611 PMC192384

[B24] Kariyama R Mitsuhata R Chow JW Clewell DB Kumon H Simple and reliable multiplex PCR assay for surveillance isolates of vancomycin-resistant enterococci J Clin Microbiol 2000 13 8 3092 3095 10921985 10.1128/jcm.38.8.3092-3095.2000 PMC87194

[B25] Shankar V Baghdayan AS Huycke MM Lindahl G Gilmore MS Infection-derived *Enterococcus faecalis* strains are enriched in *esp*, a gene encoding a novel surface protein Infect Immun 1999 13 1 193 200 9864215 10.1128/iai.67.1.193-200.1999 PMC96296

[B26] Morrison D Woodford N Barrett SP Sisson P Cookson BD DNA banding pattern polymorphism in vancomycin-resistant *Enterococcus faecium* and criteria for defining strains J Clin Microbiol 1999 13 4 1084 1091 10074530 10.1128/jcm.37.4.1084-1091.1999 PMC88653

[B27] Turabelidze D Kotetishvili M Kreger A Morris JG Jr Sulakvelidze A Improved pulsed-field gel electrophoresis for typing vancomycin-resistant enterococci J Clin Microbiol 2000 13 11 4242 4245 11060099 10.1128/jcm.38.11.4242-4245.2000 PMC87572

[B28] Tenover FC Arbeit RD Goering RV Mickelsen PA Murray BE Persing DH Swaminathan B Interpreting chromosomal DNA restriction patterns produced by pulsed-field gel electrophoresis: criteria for bacterial strain typing J Clin Microbiol 1995 13 9 2233 2239 7494007 10.1128/jcm.33.9.2233-2239.1995 PMC228385

[B29] Mullane NR Whyte P Wall PG Quinn T Fanning S Application of pulsed-field gel electrophoresis to characterise and trace the prevalence of *Enterobacter sakazakii* in an infant formula processing facility Int J Food Microbiol 2007 13 1 73 81 10.1016/j.ijfoodmicro.2006.12.036 17307267

[B30] Torres E Perez S Vindel A Rodriguez-Bano J Camba V Villanueva R Coque TM Bou G Glycopeptide-resistant *Enterococcus faecium* in a hospital in northern Spain. Molecular characterization and clinical epidemiology Enferm Infecc Microbiol Clin 2009 13 9 511 517 10.1016/j.eimc.2008.09.014 19477049

[B31] Pourakbari B Aghdam MK Mahmoudi S Ashtiani MT Sabouni F Movahedi Z Alyari AE Sadeghi RH Mamishi S High frequency of vancomycin-resistant *Enterococcus faecalis* in an Iranian referral children medical hospital Maedica 2012 13 3 201 204 23400108 PMC3566882

[B32] Werner G Klare I Fleige C Witte W Increasing rates of vancomycin resistance among *Enterococcus faecium* isolated from German hospitals between 2004 and 2006 are due to wide clonal dissemination of vancomycin-resistant enterococci and horizontal spread of vanA clusters Int J Med Microbiol 2008 13 5–6 515 527 17977789 10.1016/j.ijmm.2007.05.008

[B33] Weng PL Ramli R Shamsudin MN Cheah YK Hamat RA High Genetic Diversity of *Enterococcus faecium* and *Enterococcus faecalis* Clinical Isolates by Pulsed-Field Gel Electrophoresis and Multilocus Sequence Typing from a Hospital in Malaysia Biomed Res Int 2013 13 938937 23819125 10.1155/2013/938937 PMC3681219

[B34] Araoka H Kimura M Yoneyama A A surveillance of high-level gentamicin-resistant enterococcal bacteremia J Infect Chemother 2011 13 3 433 434 10.1007/s10156-010-0175-0 21042826

[B35] Murray BE Vancomycin-resistant enterococcal infections N Engl J Med 2000 13 10 710 721 10.1056/NEJM200003093421007 10706902

[B36] Watanabe S Kobayashi N Quinones D Nagashima S Uehara N Watanabe N Genetic diversity of enterococci harboring the high-level gentamicin resistance gene aac(6′)-Ie-aph(2″)-Ia or aph(2″)-Ie in a Japanese hospital Microb Drug Resist 2009 13 3 185 194 10.1089/mdr.2009.0917 19728776

[B37] Leavis HL Willems RJ Top J Spalburg E Mascini EM Fluit AC Hoepelman A De Neeling AJ Bonten MJ Epidemic and nonepidemic multidrug-resistant *Enterococcus faecium* Emerg Infect Dis 2003 13 9 1108 1115 10.3201/eid0909.020383 14519248 PMC3016763

[B38] Coque TM Willems R Canton R Del Campo R Baquero F High occurrence of *esp* among ampicillin-resistant and vancomycin-susceptible *Enterococcus faecium* clones from hospitalized patients J Antimicrob Chemother 2002 13 6 1035 1038 10.1093/jac/dkf229 12461029

[B39] Eaton TJ Gasson MJ A variant enterococcal surface protein Esp(fm) in *Enterococcus faecium*; distribution among food, commensal, medical, and environmental isolates FEMS Microbiol Lett 2002 13 2 269 275 10.1111/j.1574-6968.2002.tb11446.x 12435513

[B40] Dupre I Zanetti S Schito AM Fadda G Sechi LA Incidence of virulence determinants in clinical *Enterococcus faecium* and *Enterococcus faecalis* isolates collected in Sardinia (Italy) J Med Microbiol 2003 13 Pt 6 491 498 12748268 10.1099/jmm.0.05038-0

[B41] Billstrom H Lund B Sullivan A Nord CE Virulence and antimicrobial resistance in clinical *Enterococcus faecium* Int J Antimicrob Agents 2008 13 5 374 377 10.1016/j.ijantimicag.2008.04.026 18715765

[B42] Vankerckhoven V Van Autgaerden T Vael C Lammens C Chapelle S Rossi R Jabes D Goossens H Development of a multiplex PCR for the detection of *asa1*, *gelE*, *cylA*, *esp*, and *hyl* genes in enterococci and survey for virulence determinants among European hospital isolates of *Enterococcus faecium* J Clin Microbiol 2004 13 10 4473 4479 10.1128/JCM.42.10.4473-4479.2004 15472296 PMC522368

[B43] Biendo M Adjide C Castelain S Belmekki M Rousseau F Slama M Ganry O Schmit JL Eb F Molecular characterization of glycopeptide-resistant enterococci from hospitals of the picardy region (france) Int J Microbiol 2010 13 150464 21052490 10.1155/2010/150464 PMC2967830

[B44] Cha JO Jung YH Lee HR Yoo JI Lee YS Comparison of genetic epidemiology of vancomycin-resistant *Enterococcus faecium* isolates from humans and poultry J Med Microbiol 2012 13 Pt 8 1121 1128 22538996 10.1099/jmm.0.037549-0

[B45] Kuriyama T Williams DW Patel M Lewis MA Jenkins LE Hill DW Hosein IK Molecular characterization of clinical and environmental isolates of vancomycin-resistant *Enterococcus faecium* and *Enterococcus faecalis* from a teaching hospital in Wales J Med Microbiol 2003 13 Pt 9 821 827 12909661 10.1099/jmm.0.05123-0

[B46] Poh LW Rukman AW Cheah YK Norital Z Nazri AM Mariana NS Vancomycin-resistant *Enterococcus faecium* of multi locus sequence type 18 in Malaysia Med J Malaysia 2012 13 6 639 640 23770967

[B47] Willems RJ Top J Van Schaik W Leavis H Bonten M Siren J Hanage WP Corander J Restricted gene flow among hospital subpopulations of *Enterococcus faecium* MBio 2012 13 4 e00151 00112 22807567 10.1128/mBio.00151-12 PMC3413404

[B48] Werner G Coque TM Hammerum AM Hope R Hryniewicz W Johnson A Klare I Kristinsson KG Leclercq R Lester CH Emergence and spread of vancomycin resistance among enterococci in Europe Euro Surveill 2008 13 47 19021959

[B49] Freitas AR Novais C Ruiz-Garbajosa P Coque TM Peixe L Dispersion of multidrug-resistant *Enterococcus faecium* isolates belonging to major clonal complexes in different Portuguese settings Appl Environ Microbiol 2009 13 14 4904 4908 10.1128/AEM.02945-08 19447948 PMC2708421

[B50] Howden BP Holt KE Lam MM Seemann T Ballard S Coombs GW Tong SY Grayson ML Johnson PD Stinear TP Genomic insights to control the emergence of vancomycin-resistant enterococci MBio 2013 13 4 10.1128/mBio.00412-13 PMC3747580 23943759

[B51] Galloway-Pena J Roh JH Latorre M Qin X Murray BE Genomic and SNP analyses demonstrate a distant separation of the hospital and community-associated clades of *Enterococcus faecium* PLoS One 2012 13 1 e30187 10.1371/journal.pone.0030187 22291916 PMC3266884

[B52] Palmer KL Godfrey P Griggs A Kos VN Zucker J Desjardins C Cerqueira G Gevers D Walker S Wortman J Comparative genomics of enterococci: variation in *Enterococcus faecalis*, clade structure in *E. faecium*, and defining characteristics of *E. gallinarum* and *E. casseliflavus* MBio 2012 13 1 e00318 00311 22354958 10.1128/mBio.00318-11 PMC3374389

[B53] De Been M Van Schaik W Cheng L Corander J Willems RJ Recent recombination events in the core genome are associated with adaptive evolution in *Enterococcus faecium* Genome Biol Evol 2013 13 8 1524 1535 10.1093/gbe/evt111 23882129 PMC3762198

[B54] Van Schaik W Top J Riley DR Boekhorst J Vrijenhoek JE Schapendonk CM Hendrickx AP Nijman IJ Bonten MJ Tettelin H Pyrosequencing-based comparative genome analysis of the nosocomial pathogen *Enterococcus faecium* and identification of a large transferable pathogenicity island BMC Genomics 2010 13 239 10.1186/1471-2164-11-239 20398277 PMC2858755

[B55] Reuter S Ellington MJ Cartwright EJ Koser CU Torok ME Gouliouris T Harris SR Brown NM Holden MT Quail M Rapid bacterial whole-genome sequencing to enhance diagnostic and public health microbiology JAMA Intern Med 2013 13 15 1397 1404 10.1001/jamainternmed.2013.7734 23857503 PMC4001082

